# Pyramidal Neurons of the Zebrafish Tectum Receive Highly Convergent Input From Torus Longitudinalis

**DOI:** 10.3389/fnana.2021.636683

**Published:** 2021-02-03

**Authors:** Elisabeth DeMarco, Alexander L. Tesmer, Bruna Hech, Koichi Kawakami, Estuardo Robles

**Affiliations:** ^1^Department of Biological Sciences and Purdue Institute for Integrative Neuroscience, Purdue University, West Lafayette, IN, United States; ^2^Department of Gene Function, National Institute of Genetics, Mishima, Japan

**Keywords:** RRID:AB_2535849, RRID:AB_2534096, RRID:AB_371416, RRID:AB_2187677, whole brain imaging, genetic labeling, zebrafish

## Abstract

The torus longitudinalis (TL) is a midbrain structure unique to ray finned fish. Although previously implicated in orienting behaviors elicited by changes in ambient lighting, the role of TL in visual processing is not well-understood. TL is reciprocally connected to tectum and is the only known source of synaptic input to the stratum marginalis (SM) layer of tectal neuropil. Conversely, tectal pyramidal neurons (PyrNs) are the only identified tectal neuron population that forms a dendrite in SM. In this study we describe a zebrafish *gal4* transgenic that labels TL neurons that project to SM. We demonstrate that the axonal TL projection to SM in zebrafish is glutamatergic. Consistent with these axons synapsing directly onto PyrNs, SM-targeted dendrites of PyrNs contain punctate enrichments of the glutamatergic post-synaptic marker protein PSD95. Sparse genetic labeling of individual TL axons and PyrN dendrites enabled quantitative morphometric analysis that revealed (1) large, sparsely branched TL axons in SM and (2) small, densely innervated PyrN dendrites in SM. Together this unique combination of morphologies support a wiring diagram in which TL inputs to PyrNs exhibit a high degree of convergence. We propose that this convergence functions to generate large, compound visual receptive fields in PyrNs. This quantitative anatomical data will instruct future functional studies aimed at identifying the precise contribution of TL-PyrN circuitry to visual behavior.

## Introduction

The optic tectum is the largest visual brain area in zebrafish and is an important model system for examining the form and function of neural circuits mediating visually guided behavior. The neuropil of zebrafish tectum contains a highly layered structure, where retinal ganglion cell (RGC) axons with different genetic identities and visual response properties terminate in one of 9 retinorecipient tectal laminae: stratum opticum, SO; 6 sublaminae within stratum fibrosum et griseum superficiale, SFGS; stratum griseum centrale, SGC; stratum album centrale/stratum periventriculare, SAC/SPV (Xiao et al., 2005, 2011; Nikolaou et al., 2012; Kramer et al., 2019). In addition to retinorecipient layers, this neuropil also contains layers that receive input from other brain areas, including raphe nucleus, hypothalamus, and cerebellum (Heap et al., 2013, 2017; Filosa et al., 2016). Furthermore, each tectal lobe is innervated by two non-retinorecipient brain areas that contain visually-responsive neurons, nucleus isthmi and torus longitudinalis (TL). In birds, amphibians, and fish, the nucleus isthmi has been shown to mediate visual attention (Asadollahi et al., 2010, 2011; Marín et al., 2012) and recent findings suggest a similar role for nucleus isthmi in visually guided hunting behavior of larval zebrafish (Henriques et al., 2019). Less well-understood is the functional role of input from TL, a brain structure unique to ray-finned fish.

TL does not receive direct retinal input, but receives visual input indirectly via afferents from tectum and visual pretectum (Folgueira et al., 2007, 2020). Electrophysiological recordings from neurons in goldfish TL have revealed visual neurons responsive to illumination changes and non-visual neurons with activity patterns related to saccadic eye movements (Northmore, 1984). Tectum is a major target of TL efferent projections and the reciprocal circuit between TL and tectum has been studied in several fish species, including carp, trout, longnose gar, goldfish, squirrelfish, and zebrafish (Northcutt, 1982; Pérez-Pérez et al., 2003; Xue et al., 2003; Folgueira et al., 2020). In these fish species each lobe of TL selectively innervates the ipsilateral tectal lobe, forming terminal arbors in stratum marginalis (SM), the most superficial layer of the tectal neuropil. Within SM, axons from TL form synaptic contacts onto the spiny dendrites of pyramidal neurons (PyrNs; Laufer and Vanegas, 1974), a morphological class of tectal interneurons identified in both adult and larval fish (Laufer and Vanegas, 1974; Meek, 1981; Ito and Kishida, 2004; Folgueira et al., 2007, 2020; DeMarco et al., 2019). In addition to the spiny dendrite located in SM, PyrNs also form a dendritic arbor in SFGS that is a likely site of direct input from contralateral retina. Electrophysiological recordings from morphologically identified PyrNs in carp and goldfish confirmed that a majority of these neurons exhibit visually-evoked responses (Niida et al., 1980; Guthrie and Sharma, 1991).

These putative inputs suggest PyrNs may function to integrate visual input from retina with either visual or saccade-related inputs from TL. Previous anatomical studies in adult fish have suggested that ~85% of TL-derived axons in SM run parallel to each other (Laufer and Vanegas, 1974), suggesting many TL inputs have the potential to converge onto common post-synaptic PyrN dendrites. This anatomical feature has led to comparisons between the TL-derived marginal fibers and the parallel fiber inputs that synapse onto cerebellar Purkinje cell dendrites (Meek, 1992). Saccade-related TL input onto PyrNs has also been proposed to serve a function in priming visual responses during object tracking (Northmore, 2017). Within this model saccadic eye movement signals in TL are transmitted to PyrN SM dendrites and serve to sensitize PyrNs with visual receptive fields that match the predicted object location following a saccade. However, it is unknown whether visually responsive PyrNs in tectum receive visual or saccade-related input from TL. This model also assumes that saccadic TL input to PyrNs is topographically precise, which has been inferred from dense labeling experiments that did not permit visualization of single TL axon morphologies. If TL input to PyrNs is not topographically precise this would suggest saccadic input to visually responsive PyrNs may serve a more general function in sensitizing tectal circuitry during saccadic eye movements.

A more accurate wiring model necessitates a comprehensive morphological analysis of PyrN SFGS and SM dendrites as well as their inputs: RGC axon terminals in SFGS and TL axon terminals in SM. We previously identified a transgenic label for tectal PyrNs (DeMarco et al., 2019), however the number of TL and retinal inputs onto the SM and SFGS dendrites of PyrNs has not been described. Single neuron reconstructions and morphometric analysis of RGC axonal projections to every layer of the zebrafish tectum have been previously published (Robles, 2017). However, genetic single-cell labeling of TL neurons that project to the SM layer of tectum requires identification of a transgenic that labels this neuron population. We recently employed an *id2b:gal4* transgenic fish line to characterize the morphology and neurotransmitter phenotype of PyrNs in the larval zebrafish tectum (DeMarco et al., 2019). Here we identify a tissue specific transgenic, *Tg(hspGGFF23C)*, that labels TL neurons that form axonal projections to SM. Specific labeling of TL projection neurons allowed us to confirm that their neurotransmitter phenotype is glutamatergic, further supporting the idea that synapses between TL axons and PyrN dendrites are glutamatergic. The *hspGGFF23C* transgenic enabled sparse labeling of individual TL neurons innervating SM. Morphometric analysis of single TL axons revealed large, sparsely branched axon arbors that occupied more than 80% of the total SM area. In contrast, post-synaptic PyrN dendrites formed in SM occupied synaptic territories twenty times smaller than those of TL axons. This unique combination of pre- and post-synaptic morphologies suggests a high degree of convergence at the TL-PyrN connection, which has important implications for the functional role of this circuit in visual processing.

## Results

### *hspGGFF23C* Transgenic Labels TL Neurons That Form a Neurite Projection to the Tectal Neuropil

**Figure 1:**
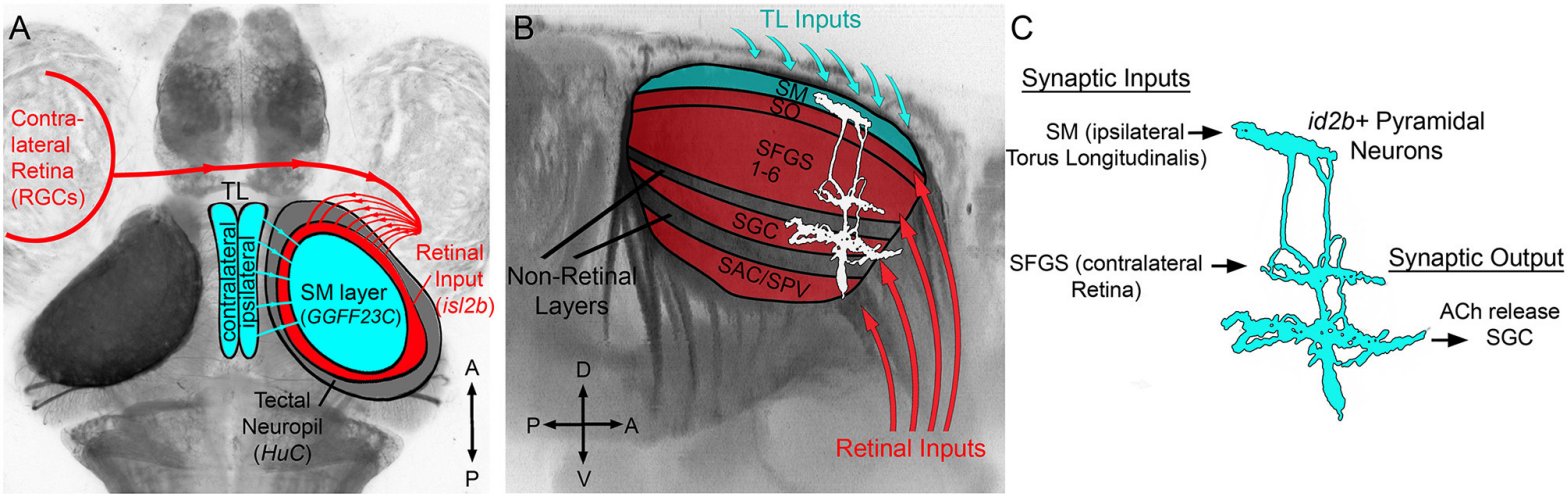
Overview of TL-tectum circuit and PyrN connectivity. **(A)** Dorsal view fluorescence image of a Tg(*HuC:lynTagRFP-t)* larval brain in which all axon tracts and neuropil areas are fluorescently labeled, with superimposed schematic of the brain regions examined in this study. Connectivity of TL-tectum circuitry: ipsilateral TL forms an axonal projection terminating in the SM layer of the tectal neuropil. The contralateral retina forms an axonal projection terminating in tectal layers distinct from SM. **(B)** Higher magnification side view of the midbrain of brain volume in **(A)**. Overlayed schematic depicts the relative laminar positions of retinal input layers relative to SM, other non-retinorecipient neuropil layers, and PyrN stratification pattern. **(C)** Putative connectivity of *id2b:gal4* positive PyrNs. Based on laminar position of dendrites PyrNs most likely receive TL-derived inputs on their SM dendrite and retinal inputs on their SFGS dendrite. PyrN axon stratifies within the SGC layer.

**Figure 2:**
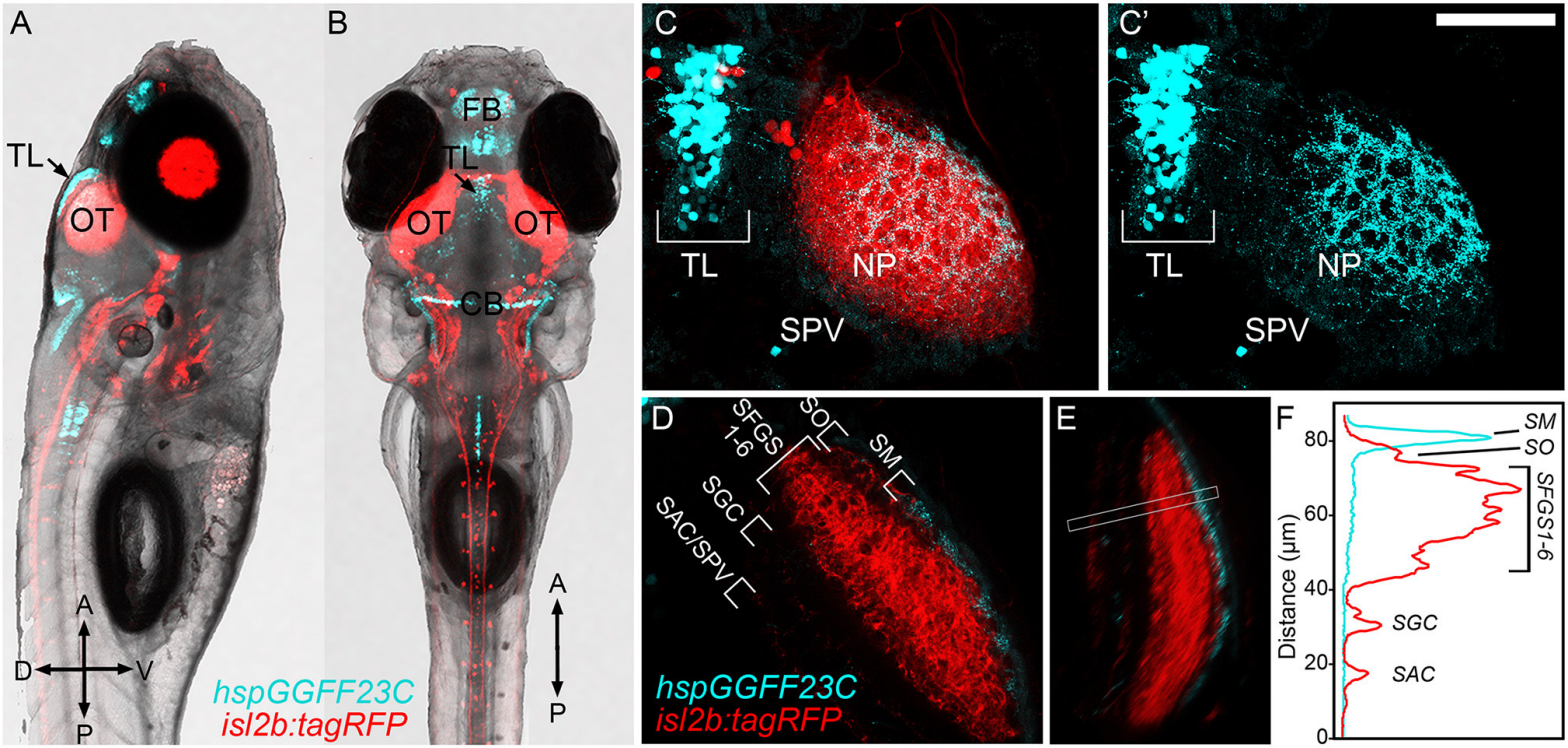
*hspGGFF23C* transgenic labels TL neurons that project to SM. **(A,B)** Low-resolution confocal images of 7 dpf triple transgenic *Tg(hspGGFF23C,uas:egfp,isl2b:tagRFP)* larva from side- and dorsal-view. **(C)** Higher magnification maximum projection images of TL and one tectal lobe of larva in **(A,B)**. Merged fluorescence channel shown in **(C)**, *hspGGFF23C*-driven EGFP channel shown in **(C′)**. Note presence of EGFP-positive cells in TL and neurite plexus formed in tectal neuropil and lack of labeling in SPV layer of tectum. **(D)** Single confocal image through tectal neuropil. Note discrete EGFP labeling in the superficial SM layer of tectal neuropil. **(E)** Rotated side-view of image volume in **(D)**, orientation orthogonal to the neuropil layers. **(F)** Fluorescence intensity profile measurement along line indicated by box in **(E)**. Note peak in EGFP signal in the superficial SM layer. Scale bar: 300 μm in **(A,B)**, 80 μm in **(C)**, and 60 μm in **(D,E)**.

We previously identified *id2b*-positive PyrNs as cholinergic tectal interneurons that forms two dendritic arbors: one in SM that receives TL input and the other in SFGS that receives RGC input from contralateral retina (Figure 1; DeMarco et al., 2019). In order to better define TL-PyrN connectivity we sought to identify a transgenic that specifically labels TL inputs to SM. As part of a large-scale Gal4 enhancer trap screen (Asakawa et al., 2008), the *Tg(hspGFF23C)* line was previously identified as a transgenic with inheritable transgene expression in the nervous system. Generation of *Tg(hspGGFF23C,uas:egfp,isl2b:tagRFP)* triple transgenic larvae allowed us to use EGFP as a fluorescence reporter for gal4 expression in addition to RFP expression in all RGCs. Use of *isl2b:tagRFP* to drive RFP expression in all RGCs permitted clear visualization of the optic nerve and neuropil region of tectum (Figures 2A,B). *hspGGFF23C*-driven EGFP reporter expression revealed cell labeling in the forebrain and cerebellum as early as 3 days post-fertilization (data not shown). Midbrain EGFP expression was clearly detectable by 5 dpf and restricted to a subset of neurons located in TL (Figures 2A,B). Higher resolution imaging of the midbrain of *Tg(hspGGFF23c:gal4,uas:egfp, isl2b:tagRFP)* larvae revealed that, in addition to cell bodies within the TL, EGFP expression also labeled a dense plexus formed by TL neurites in tectum (Figure 2C). This EGFP-positive neurite plexus innervated the SM layer of the tectal neuropil, superficial to the RGC-derived layers labeled by *isl2b:tagRFP* (Figures 2D–F). These findings identify the *hspGGFF23C* transgene as a marker for TL neurons that form a neurite projection terminating in the SM layer of tectum.

### The Projection From TL to Tectum Is Glutamatergic

**Figure 3:**
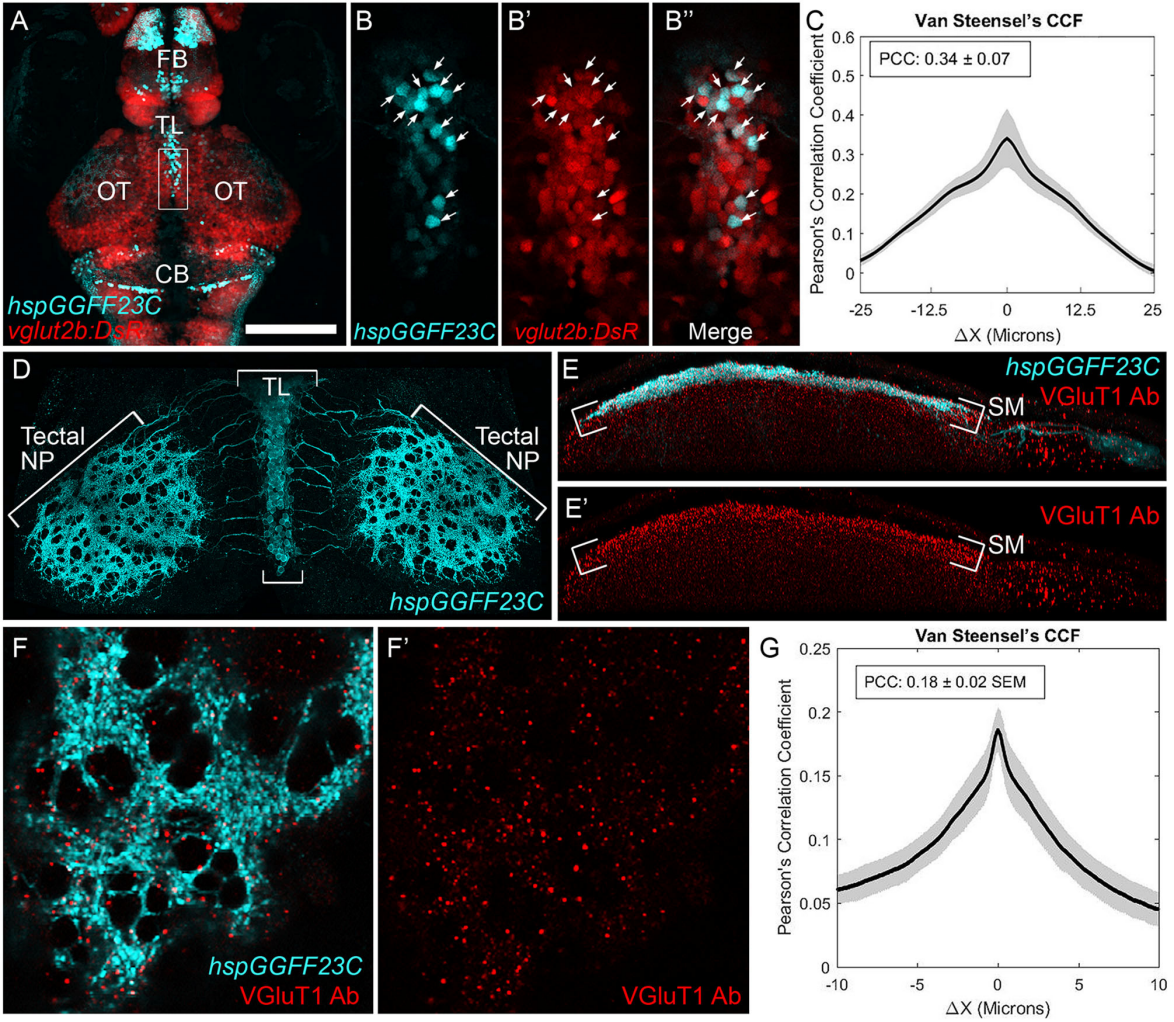
Axonal projection from TL to tectum is glutamatergic. **(A)** Dorsal view confocal image of EGFP and DsRed fluorescence in the brain of a Tg(*hspGGFF23C,uas:egfp,vglut2b:rfp)* larva. **(B)** Magnified views of region indicated by box in **(A)** showing the EGFP **(B)**, DsRed **(B′)**, and merged **(B^′′^)** fluorescence channels. Arrows indicate neurons with EGFP and DsRed colocalization. **(C)** Van Steensel's cross-correlation functions of EGFP and DsRed overlays. Local maxima of Pearson's Coefficient (PC) near ΔX = 0 indicates colocalization. *N* = 10 larvae. **(D)** Dorsal view confocal image of the tectum in a 7 dpf Tg(*hspGGFF23C,uas:egfp)* larva fixed and immunofluorescently stained with anti-EGFP antibody. Note dense plexus of EGFP-positive axons in SM. **(E)** Side-view of a single tectal lobe from tectum in **(D)**, including immunofluorescent staining with anti-VGluT1 antibody in red channel. Note superficial EGFP labeling in SM layer and punctate VGluT1 immunofluorescence. Note increased density of puncta in SM layer. **(F)** High-resolution dorsal view of a SM subregion within tectum in **(D)**. Note overlap between VGluT1 puncta and EGFP-positive plexus and lack of VGluT1 labeling in EGFP-negative regions. **(G)** Van Steensel's cross-correlation functions of EGFP and DsRed overlays for image in **(F′)**. Local maxima of Pearson's Coefficient (PC) near ΔX = 0 indicates colocalization. *N* = 4 larvae. Scale bar: 150 μm in **(A)**, 30 μm in **(B,E)**, 75 μm in **(D)**, and 15 μm in **(F)**.

Previous electrophysiological and immunocytochemical studies in adult fish have suggested that the projection from TL to SM is excitatory (Vanegas et al., 1979) and glutamatergic (Kageyama and Meyer, 1989). To confirm that this projection in the larval zebrafish is also glutamatergic, we generated triple transgenic *Tg(hspGGFF23C,uas:egfp,vglut2b:RFP)* larvae in which glutamatergic neurons are marked with red fluorescence (Figures 3A,B; Satou et al., 2013; DeMarco et al., 2019). Using 3D-colocalization analysis, we found *hspGGFF23C* labeled an average of 64.8 ± 4.7 neurons in TL. A majority of these neurons were also positive for RFP expression (86.8 ± 8.8%, *n* = 10 larvae). Analysis of the RFP and EGFP fluorescence signals in these images revealed a Pearson's Correlation Coefficient (PCC) of 0.34 ± 0.07 (Figure 3C). Furthermore, as demonstrated by the Van Steensel's Cross Correlation Function (CCF; which shows how the correlation between the two image color channels changes as they are shifted relative to each other), there is a peak at ΔX = 0 and the PCC drops off markedly as ΔX exceeds ±4 μm, which is approximately the radius of the EGFP-positive neurons in TL. These findings demonstrate that the *hspGGFF23C* transgene labels ~65 neurons in the larval TL and the majority of these are glutamatergic.

To provide additional evidence that the synapse between TL and tectum is glutamatergic, we examined the distribution of vesicular glutamate transporter 1 (VGluT1) protein within the SM layer of tectum using an antibody that recognizes zebrafish VGluT1 (Gao et al., 2018). Although TL neurons projecting to OT are labeled by the *vglut2b:RFP* transgene, in several systems VGluT1 and VGLUT2 proteins are coexpressed in the same neurons (Hisano et al., 2002; Hioki et al., 2003). Immunofluorescent antibody staining of *hspGGFF23C*-driven EGFP labeled the dense neurite plexus formed by TL axons in SM (Figure 3D). VGluT1 antibody staining revealed the SM layer of tectum to contain a high density of VGluT1 immunofluorescence (Figure 3E). High resolution imaging of subregions of SM in these specimens allowed us to examine the degree of colocalization between the punctate VGluT1 label and the *hspGGFF23C* neurite plexus. As shown in Figure 3F, this plexus contained circular regions devoid of EGFP-labeled axons, which are most likely where axons from TL were diverted by neuronal cell bodies located in the neuropil (e.g., superficial interneurons; Del Bene et al., 2010). Notably, VGluT1 immunofluorescence in SM was largely excluded from regions devoid of axons, suggesting that this labeling is specific and marks presynaptic specializations formed by TL axons in SM. Furthermore, although the PCC for these specimens was low due to the sparseness of the VGluT1 label, the Van Steensel's CCF exhibited a sharp peak at ΔX = 0 and the PCC dropped off markedly as ΔX varied from the origin (Figure 3G). Together, these data confirm previous findings that the neurite projection from TL to SM is axonal and utilizes glutamate as an excitatory neurotransmitter.

### PyrN Dendrites in SM Form PSD95-Positive Post-synaptic Specializations

**Figure 4:**
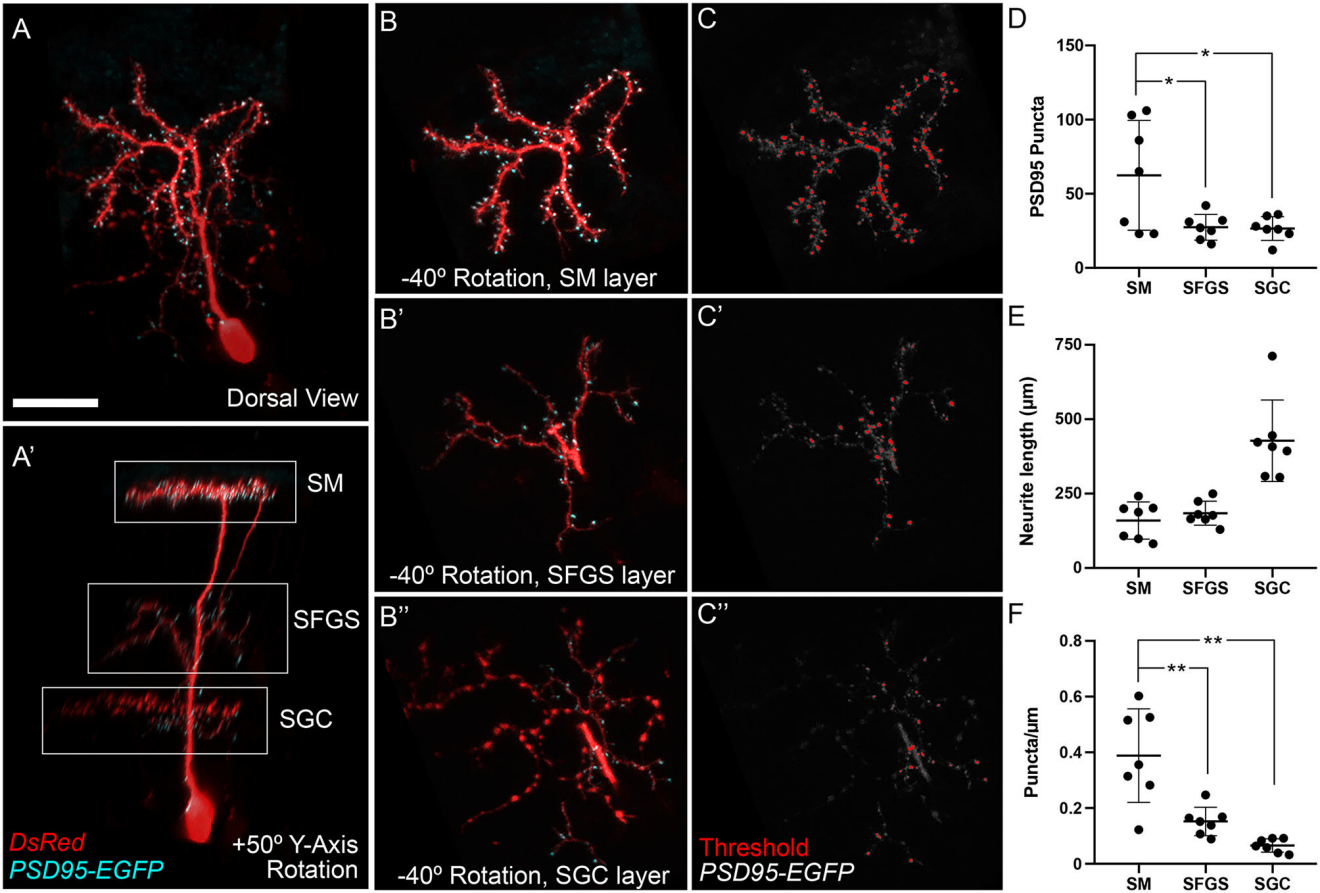
PSD95-positive post-synaptic specializations in PyrN dendrites. **(A)** Maximum projection of PSD95-EGFP and DsRed expression in an isolated *id2b:gal4* positive PyrN. Note discrete enrichments of PSD95-EGFP relative to cytosolic DsRed label. **(A′)** +50° Y-axis rotated view of image volume in **(A)**. SM, SFGS, and SGC dendritic portions are indicated by boxed regions. Note presence of PSD95-EGFP puncta in SM, SFGS, and SGC layer dendrites. **(B)** −40° Y-axis rotated views of isolated subvolumes from boxed regions in **(A′)** showing PSD95-EGFP and DsRed expression in the SM, SFGS, and SGC dendrites. **(C)** Threshold images of PSD95-EGFP channel in rotated subvolumes in **(B)** as an example of threshold values used for automated particle analysis (puncta counting). **(D–F)** Quantification of PSD95-EGFP puncta number, neurite length, and puncta density for SM, SFGS, and SGC dendrite subregions in 7 fully reconstructed PyrNs obtained from 7 larvae. Scale bar: 20 μm in **(A)** and 15 μm in **(B,C)**. *p < 0.05; **p < 0.001; ANOVA with Tukey's *post-hoc* test.

In our initial characterization of PyrNs we determined that the SM-targeted neurite was dendritic, based on a lack of presynaptic specializations containing synaptophysin (DeMarco et al., 2019). To confirm that the PyrN arbor formed in SM is dendritic and contains glutamatergic post-synaptic specializations, we used embryo injection to sparsely label PyrNs via expression of a PSD95-EGFP fusion protein and a cytosolic DsRed label (Niell et al., 2004). PSD-95 is a protein component of the post-synaptic density at glutamatergic synapses (reviewed by Sheng and Kim, 2011). Previous studies have demonstrated that exogenous overexpression of PSD95 fusion proteins can disrupt normal neuronal development by promoting excess synapse formation (Cane et al., 2014; Chen et al., 2015). We also found injection of *uas:psd95egfp:dsred* DNA was highly toxic, resulting in a reduced incidence of cell labeling, most likely due to death of neurons expressing high levels of PSD95. When labeled PyrNs were observed, these occasionally possessed simple morphologies and reduced neurite lengths compared to control neurons expressing a membrane-targeted EGFP (data not shown). In spite of these difficulties, we obtained seven neurons in 6 dpf *id2b:gal4* larvae with detectable levels of PSD95-EGFP and normal PyrN morphologies. As shown in Figure 4A, when PyrNs are imaged in the native orientation (dorsal view, larva mounted brain side up) PyrN neurite stratifications are not visible. Rotating +50° around the Y-axis clearly reveals the three distinct arbors formed in the SM, SFGS, and SGC layers of the tectal neuropil (Figure 4A′). Combining 3D visualization with a 3D clipping tool allowed us to generate image subvolumes containing a single neurite stratification (SM, SFGS, or SGC; Figures 4B–B^′′^). Consistent with our previous findings, SM and SFGS arbors contained punctate enrichments of PSD95-EGFP relative to the cytosolic DsRed label. Every PSD95-EGFP-labeled PyrN also contained sparse punctate enrichments of PSD95-EGFP within the SGC-targeted neurite we previously assumed was purely axonal (Figures 4A′,B^′′^,C^′′^; 7 of 7 neurons). The SGC arbor contained PSD95-EGFP puncta primarily within branches of the arbor lacking varicosities, suggesting this neurite contains both axonal (presynaptic) and dendritic (post-synaptic) branches. We used these PyrN layer subvolumes to count PSD95-EGFP puncta and measure neurite lengths (Figures 4D–F). This analysis revealed greater puncta number (*P*-values of 0.0236 and 0.0203) and puncta density (*P* < 0.005) for the SM dendrite compared to SFGS and SGC arbors. Together these data confirm that the PyrN SM dendrite contains excitatory post-synaptic specializations, consistent with their role as the post-synaptic targets of glutamatergic TL axons.

### TL Axons in SM Form Large, Sparsely Branched Arbors

We set out to determine how the dense axonal plexus in SM (see Figures 3D,F) is formed by the structures of individual TL neurons. One possibility is that individual TL axons form narrow arbors at discrete positions along the anteroposterior axis, forming synaptic territories that exhibit a low degree of overlap with neighboring axons. This would support wiring models generated from anatomical studies in adult fish tectum (Laufer and Vanegas, 1974; Meek, 1992) and resemble circuit architectures where neurites form tiled patterns to maximize coverage while minimizing overlap. This type of circuit motif has been observed in many sensory systems and may represent an ideal solution to minimize information redundancy (Grueber and Sagasti, 2010). An alternative scenario is one in which individual TL axons form large, sparsely branched arbors with a large degree of spatial overlap. This circuit motif could represent an ideal solution to maximize convergence of multiple TL inputs onto single post-synaptic PyrNs. In either scenario, this growth pattern, when viewed as a population, could yield the dense axon plexus observed in the *hspGGFF23C* transgenic.

**Figure 5:**
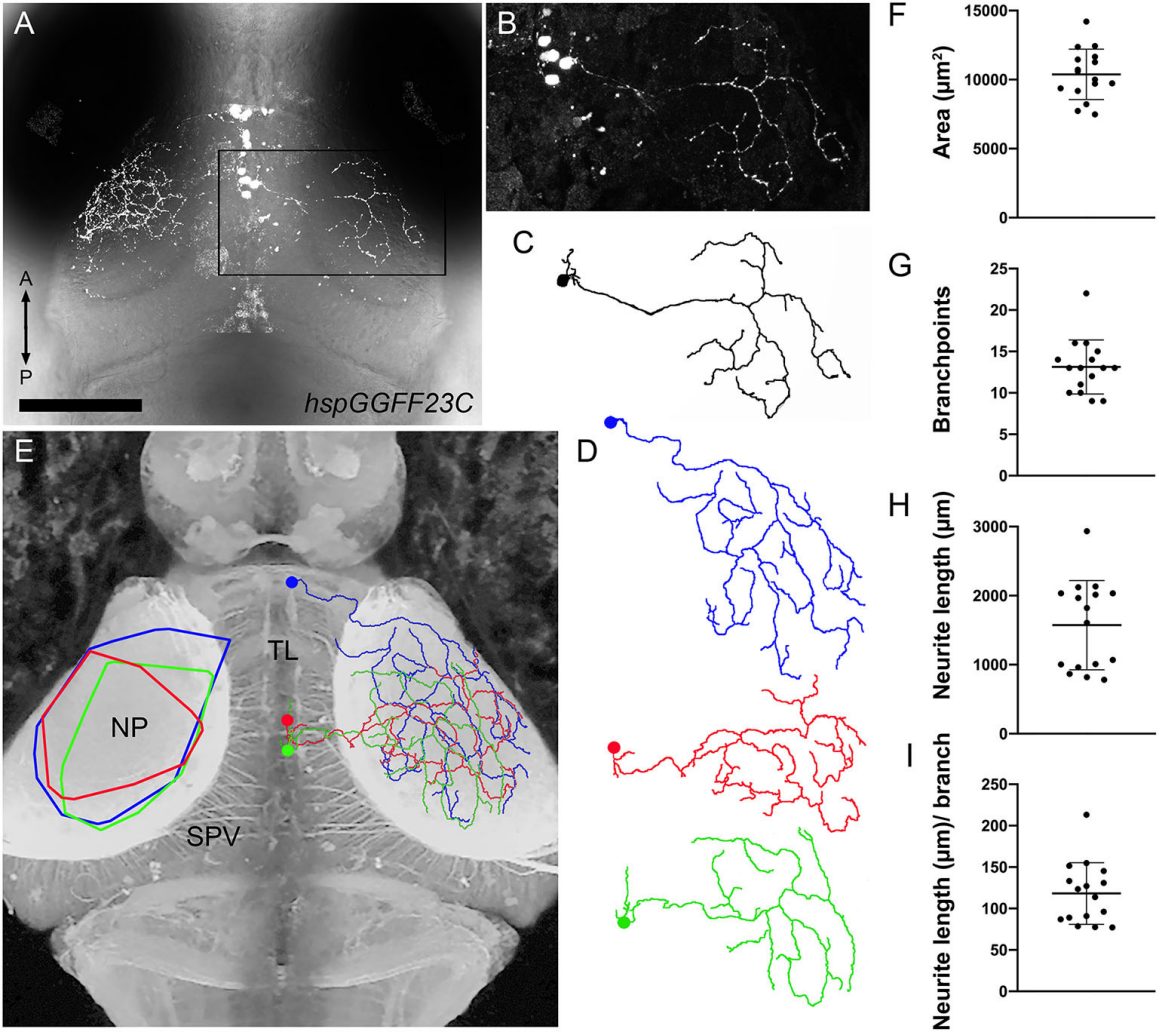
Morphology of individual TL axons innervating SM. **(A)** Dorsal view confocal image of the midbrain from a 7 dpf Tg(*hspGGFF23C:gal4,uas:NTR-mCherry)* larva containing several labeled neurons in the left TL lobe and a single neuron in the right TL lobe, indicated by boxed region. **(B)** Magnified view of boxed region in **(A)**. **(C)** Skeletonized tracing of SMTL neuron in **(B)**. Note large, sparsely branched arbor. **(D)** Skeletonized tracings of three SMTL neurons. Note large, sparsely branched arbor and small dendritic arbors within TL. **(E)** Dorsal view inverted fluorescence image of the brain of a Tg(*HuC:lynTagRFP-t)* larva. Overlayed on the right tectum are reconstructed and appropriately scaled tracings of three SMTL neurons in **(D)** that innervated SM. Note that the three SMTL neurons vary in their cell body position within TL, but all are located in ipsilateral TL. Overlayed on the left tectum are convex polygons that summarize the SM area spanned by each of the three SMTL axons shown at right. **(F–I)** Quantification of retinotopic area, branchpoint number, total neurite length, and branching density for 16 reconstructed SMTL axons obtained from 12 larvae. Scale bar: 120 μm in **(A)**, 80 μm in **(B–D)**, and 90 μm in **(E)**.

To generate sparse mosaic labeling, we initially performed injections of *uas-egfp-caax* plasmid DNA into early stage *Tg(hspGGFF23C,uas:NTR-mCherry)* embryos (DeMarco et al., 2019). However, this approach did not reliably yield labeling of TL neurons, possibly due to weak Gal4 expression in TL at early stages of development. However, visual inspection of injected larvae for sparse EGFP-caax expression revealed that neuronal labeling in *Tg(hspGGFF23C,uas:NTR-mCherry)* larvae was highly variegated. In ~10% of mCherry-positive larvae, isolated TL neurons were observed in one or both lobes of TL (Figures 5A,B). This strategy allowed us to accurately reconstruct 16 individual TL neurons. Every neuron that was labeled innervated the SM layer of tectum, suggesting that the *hspGGFF23C* transgene specifically labels a single neuron type we have named the SM-projecting TL neuron (SMTL). In each case SMTL axons innervated ipsilateral tectum, consistent with previous findings (Northcutt, 1982; Pérez-Pérez et al., 2003; Xue et al., 2003; Folgueira et al., 2020). Once within the tectal neuropil, SMTL axons formed large, sparsely branched arbors occupying a large portion of the SM layer (Figures 5C–E). Semi-automated segmentation and quantitative morphometry revealed that SMTL axon arbors in SM had an average area of 10382.4 ± 527.5 μm^2^ and formed a branchpoint every 118.1 ± 9.3 μm of neurite length (Figures 5F–I). For four of these neurons it was also possible to reconstruct the morphology of their dendrites, which were small, simple, and restricted to the same TL lobe as the cell body (Figures 5C,D). The large synaptic territories of SMTL axons support a model in which a high degree of spatial overlap reflects convergence of TL-derived inputs onto PyrN dendrites.

### TL Inputs to PyrNs Exhibit a High Degree of Convergence

**Figure 6:**
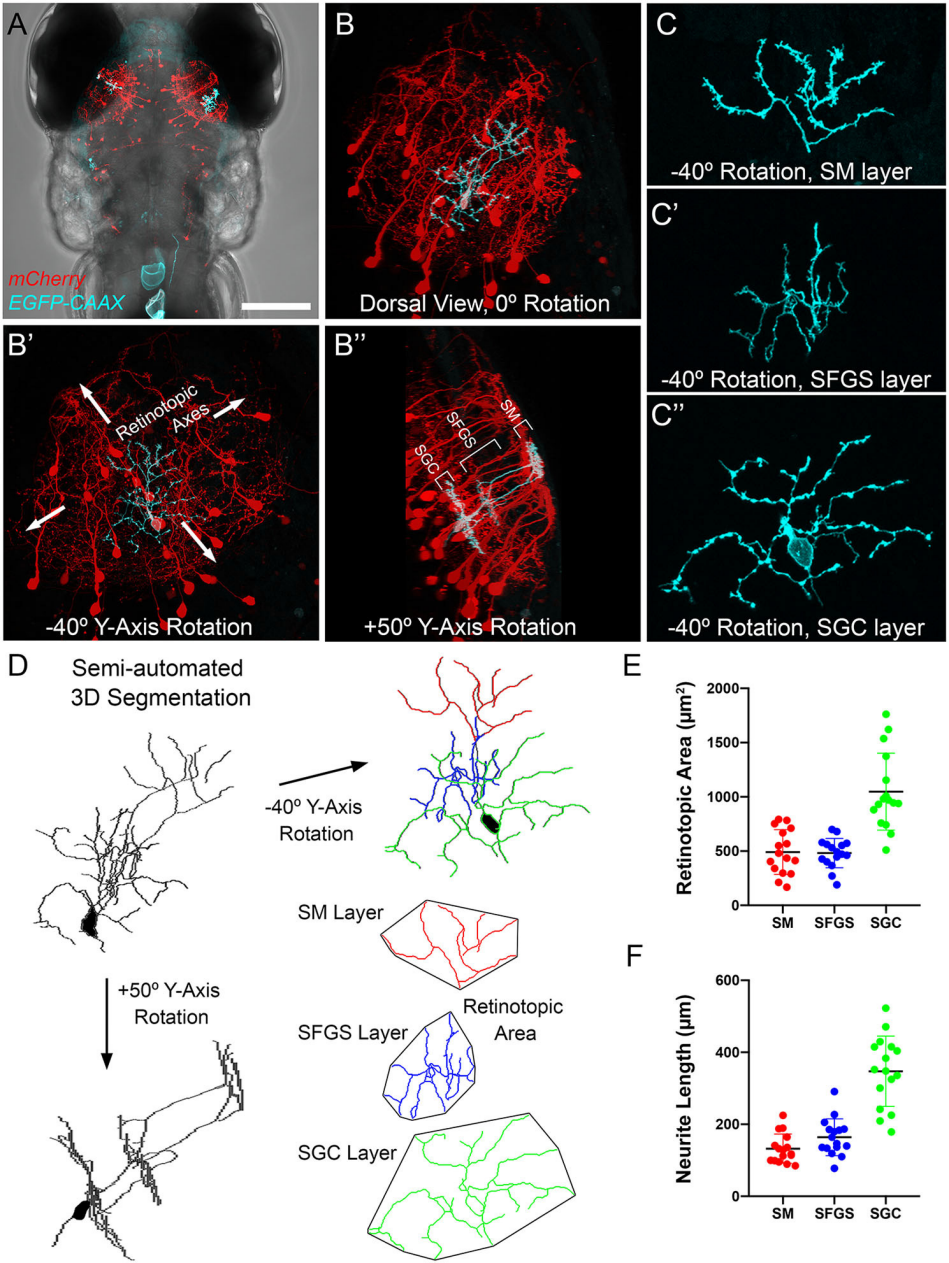
Morphometry of PyrN arbors formed in SM, SFGS, and SGC layers of tectum. **(A)** Dorsal view, whole-brain confocal image volume of a 7 dpf double transgenic *Tg(id2b:gal4,uas:NTR-mCherry)* larva injected at the embryo stage with a *uas:egfp-caax* plasmid to yield sparse genetic labeling. Note single EGFP-labeled neuron in each tectal lobe. **(B)** Higher magnification maximum projection of neuron labeled in right tectal lobe of larva in **(A)**. Projection is shown from dorsal view, 0° Y-Axis rotation. Maximum projection of same neuron rotated −40° about the Y-axis, an orientation parallel to the tectal layers **(B′)**. Maximum projection of same neuron rotated +50° about the Y-axis, an orientation orthogonal to the tectal layers **(B^′′^)**. Note clearly stratified neurite morphology with arbors in SM, SFGS, and SGC layers of tectal neuropil. **(C)** High magnification views of isolated subvolumes of the SM **(C)**, SFGS **(C′)**, and SGC **(C^′′^)** layer dendrites of neuron in B shown with −40° Y-axis rotation used to calculate retinotopic areas. **(D)** Workflow for morphological segmentation and measurement of retinotopic areas for PyrN neurite subvolumes. Semi-automated 3D segmentation produces skeletonized tracings used for neurite length and retinotopic area measurements. For direct comparison between raw images and tracings, note that skeletonized tracing was obtained from PyrN shown in **(B,C)**. **(E,F)** Quantification of retinotopic area and neurite length measurements for the different PyrN neurite subvolumes: SM, SFGS, and SGC. Scale bar: 200 μm in **(A)**, 50 μm in **(B)**, 15 μm in **(C)**, and 20 μm in **(D)**.

Single neuron reconstructions of SMTL axons innervating tectum suggest synaptic territories with a high degree of spatial overlap. To explore the plausibility of a scenario where a large proportion of SMTL axons synapse onto a single PyrN, we compared the relative sizes of pre- and post-synaptic axonal territories in SM and SFGS. To visualize PyrN single cell morphology, we generated larvae with mosaic genetic labeling of a membrane-targeted EGFP by injection of *uas:egfp-caax* plasmid DNA into early stage *Tg(id2b:gal4,uas:NTR-mCherry)* double transgenic embryos (DeMarco et al., 2019). Image volumes with single PyrNs (Figures 6A,B) were rotated +50° around the Y-axis to confirm the characteristic stratified morphology of PyrNs (Figure 6B^′′^) and create 3D subvolumes of individual SM, SFGS, and SGC neurite arbors. Rotation of these subvolumes −40° around the Y-axis yielded a view orthogonal to the stratified side-view (Figures 6C–C^′′^). This orientation was used to measure retinotopic areas and neurite lengths for individual PyrN arbors in SM, SFGS, and SGC (Figures 6D–F) using a convex polygon connecting the outermost branch tips (Teeter and Stevens, 2011). These analyses revealed that SM PyrN dendritic arbors occupied areas within SM that were ~20-fold smaller than those occupied by TL axons (491.7 ± 51.4 μm^2^ vs. 10382.4 ± 527.5 μm^2^).

**Figure 7:**
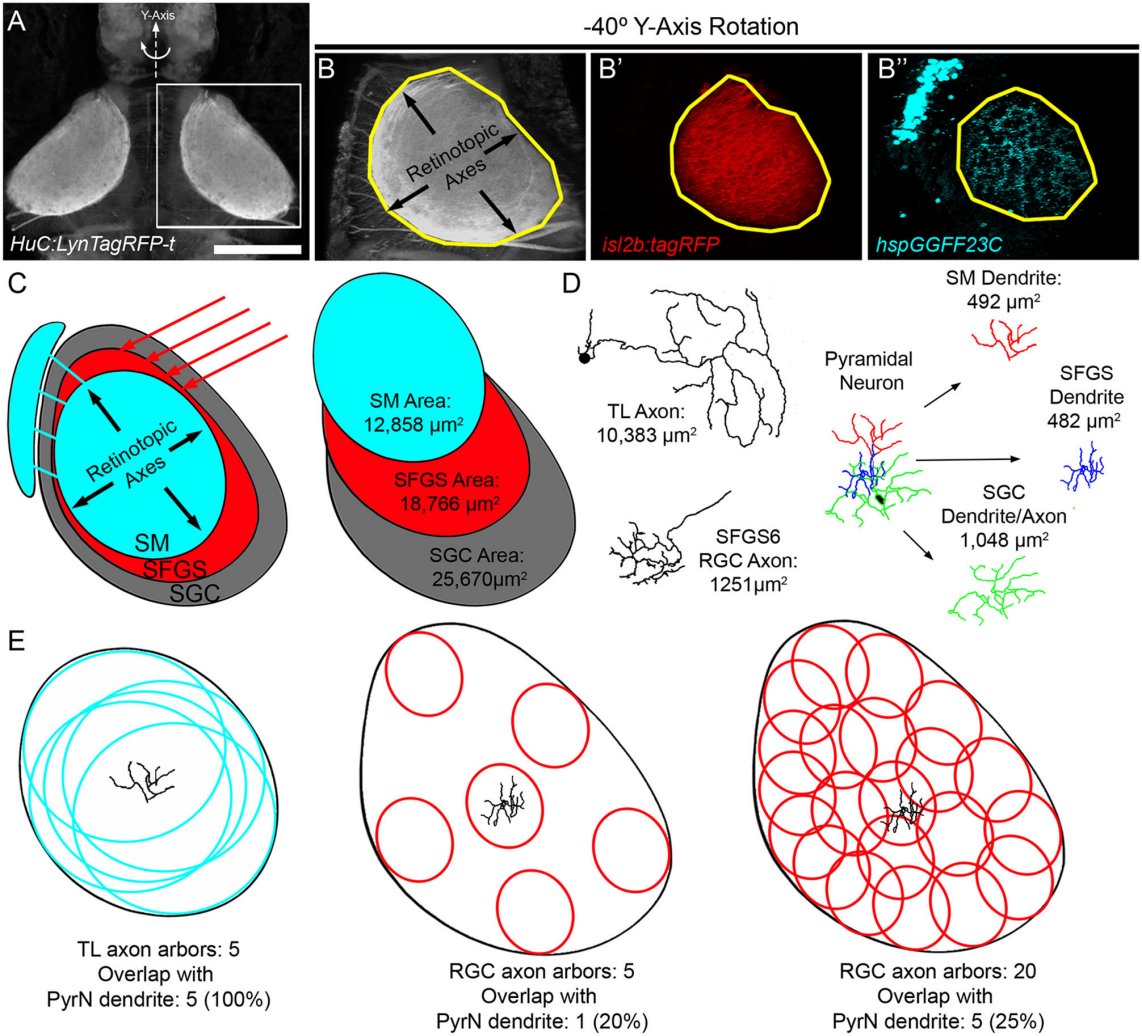
Evidence for high degree of convergence from TL inputs to PyrNs. **(A)** Maximum projection image of fluorescence in a 7 dpf Tg(*HuC:lynTagRFP-t)* larva. **(B)** Maximum projection of boxed subregion in **(A)** rotated −40° about the Y-axis, an orientation orthogonal to the tectal layers used for retinotopic area measurements. Maximum projection of equivalent subregions in a 7 dpf *Tg(hspGGFF23C,uas:egfp,isl2b:TagRFP)* larva rotated −40° about the Y-axis. Yellow polygon drawn to assist in comparison of area differences between the three images. **(C)** Schematic at left summarizes tectal inputs from TL-recipient SM layer (cyan), retinorecipient layers (red), and total tectal neuropil area. Schematic at right summarizes area measurements for these different neuropil layers. **(D)** Skeletonized tracings and average retinotopic areas for TL axons, RGC axons, and PyrN neurite subregions. **(E)** Implications of size disparity between TL axon arbors and retinal axon arbors in tectum. TL axon arbor areas are depicted as cyan circles and RGC axon arbor areas are red ovals. Due to the large size of TL arbors, a PyrN dendrite at the center of SM has the potential to contact all TL inputs. Conversely, the small relative size of RGC axons innervating SFGS means that a PyrN dendrite at the center of SFGS has the potential to contact only a small fraction of retinal inputs to SFGS. Due to the small size of both pre- and post-synaptic elements this remains true even as the number of RGC inputs increases. Scale bar: 200 μm in **(A)** and 100 μm in **(B)**.

The wiring geometry of SMTL axons and PyrN dendrites is consistent with a model in which many TL axons synapse onto each PyrN. Is this wiring geometry fundamentally different than the connection between PyrNs and RGCs axons from contralateral retina? To perform this comparison we used morphological data from SMTL axons (Figure 5), PyrN dendrites (Figure 6), and published area measurements of single SFGS RGC axons (Robles et al., 2013). Due to the ovoid shape of the tectal neuropil, the area occupied by SM (using *hspGGFF23C* as a marker) is smaller than the area of SFGS (using i*sl2b:TagRFP* as a marker; Figures 7A–C). In turn, both of these are smaller than the total neuropil area (using *HuC:lynTagRFP-t* as a marker). For retinotopic area measurements, representative image volumes were rotated −40° around the Y-axis, yielding an orientation roughly orthogonal to the synaptic layers (Figure 7D). Area measurements for SM, SFGS, and SGC neuropil layers are presented in Table 1. These measurements allowed us to express neurite arbor territories as a fraction of the area for its corresponding neuropil layer. For example, each SMTL axon typically occupied 81.3% of the SM area, whereas each PyrN dendrite arbor occupied only 3.8% of SM. By comparison, RGC axon arbors innervating SFGS6 occupied 6.7% of SFGS and PyrN dendrites in SFGS occupied 2.6%. The large area spanned by SMTL axons in SM makes it feasible for a large fraction of these axons to overlap with a single PyrN SM dendrite and form synapses on it (Figure 7E). In contrast, due to the small size of both RGC axon arbors and PyrN dendrites in SFGS a single PyrN-SFGS dendrite has the potential to sample a small fraction of RGC inputs to SFGS. Due to the small areas of RGC inputs and PyrN dendrites, as RGC axon input number increases, the number of potential inputs in our model will rise yet remain a small fraction of the total (Figure 7E).

**Table 1:** Cell type-specific morphological analysis of arbor area in tectal neuropil.

	**SM**			**SFGS**			**Tectal**	
**Label**	**23c**	**23c single axon**	**PyrN-SM**	**isl2b**	RGC-SFGS6^*^	**PyrN-SFGS**	**HuC**	**PyrN-SGC**
Area (μm^2^)	12857.7 ± 240.8	10382.4 ± 527.5	491.7 ± 51.4	18766.3 ± 135.2	1251.2 ± 130.7	481.7 ± 33.7	25670.4 ± 719.2	1048.2 ± 88.7
*N*	8	16	16	8	21	16	8	16
Percent of total	100%	81.3%	3.8%	100%	6.7%	2.6%	100%	4.1%

*SM measurements indicate area occupied by the axonal plexus (23c), single TL axons, and single PyrN SM dendrites. SFGS measurements indicate area occupied by RGC terminal arbors (isl2b), single RGC axons, and single PyrN SM dendrites. Area measurements for single PyrN SGC dendrites expressed as percentage of tectal neuropil area determined by pan-neuronal HuC labeling. Note the large axonal territories of single TL axons in SM. All data are presented as mean ± SEM*.

* *Data previously published in Robles et al. (2013)*.

## Discussion

Single cell labeling of presynaptic TL neurons and post-synaptic PyrNs enabled a detailed examination of how their morphologies constrain potential wiring diagrams. TL is the only known source of synaptic input to SM, and direct synapses have been confirmed in other fish species (Laufer and Vanegas, 1974). Our findings demonstrate that SMTL axons are glutamatergic and PyrN dendrites in SM contain glutamatergic post-synaptic specializations, direct evidence that SMTLs provide synaptic input to PyrNs in the larval zebrafish. Single cell reconstructions of SMTL morphologies revealed axons that innervated the ipsilateral tectum, where they form extremely large arbors in the SM neuropil layer. After controlling for differences in layer areas, SMTL axons exhibited synaptic territories 12 times larger than RGC axons (Robles et al., 2013). This large arbor correlated with reduced branch density, a conserved feature of neural circuits across several species (Teeter and Stevens, 2011). In the TL-PyrN circuit, synaptic area is also inversely related to the number of inputs: the total number of RGCs innervating SFGS6 is ~450 at this larval stage (Robles et al., 2013, 2014), 13 times greater than the number of SM-projecting neurons per TL lobe (~35). Despite this vast difference in number, the small size of RGC axons and PyrN SFGS dendrites ensures that retinal input onto each PyrN is from a small number of RGCs with similar cell body locations in retina. In contrast, a single PyrN SM dendrite in central tectum could easily have a synaptic territory that overlaps with all inputs from ipsilateral TL. This vastly different potential for convergence from RGC and TL inputs is further supported by SM PyrN dendrites containing twice as many post-synaptic specializations as SFGS PyrN dendrites (Figure 4). The combination of large, sparsely branched SMTL axons and densely innervated SM dendrites (Figure 4) indicates that PyrNs are most likely innervated by a large number of SMTLs. Combined with the largely overlapping TL axons, a scenario is plausible where a single PyrN receives 60 synapses from all 35 ipsilateral SMTLs while receiving only 25 inputs from 25 RGCs (18% of SFGS RGC axons) on its SFGS dendrite (see Figure 7). However, in many retinorecipient brain areas, single RGCs make multiple synaptic contacts onto a single post-synaptic neuron (Hamos et al., 1987; Hong et al., 2014; Hammer et al., 2015), suggesting the actual number of RGCs forming these 25 synapses onto each PyrN could be lower.

What are the functional implications of this putative TL-PyrN wiring geometry? Previous electrophysiological single neuron recordings from carp and goldfish tectum have shown that PyrNs can exhibit responses to either positive (ON) or negative (OFF) contrast steps. Regardless of contrast sensitivity, a common feature of PyrNs was large receptive fields (RFs) spanning more than 70° of the visual field (Niida et al., 1980; Guthrie and Sharma, 1991). If PyrNs of the larval zebrafish have similarly large RFs, the small retinotopic area of their dendrites would seem at odds with them receiving inputs from large numbers of RGCs. In mouse superior colliculus (tectum), neurons with large visual RFs also have wide, sparsely branched dendrites capable of directly sampling many RGC inputs within the superficial retinal input layers (Gale and Murphy, 2014). We propose that the unique wiring geometry of the TL-PyrN circuit serves to degrade topographic precision to generate PyrNs with large visual RFs. TL receives visual input from tectum via TLPNs, visually responsive tectal neurons with small dendrites in SFGS that project to TL (Robles et al., 2020). Single cell labeling of TLPNs has confirmed that their axons form a coarse topographic projection to TL (DeMarco et al., 2019). These observations suggest that the larval zebrafish TL contains an orderly map of visual space, which has been previously demonstrated in adult goldfish TL (Northmore, 1984). If the large degree of spatial overlap between TL axons reflects convergence, this would result in each PyrN receiving inputs from SMTLs with RFs spanning separate regions of visual space. If SM input is a strong determinant of PyrN spiking, this patchwork of input RFs would generate large, compound RFs in PyrNs. Consistent with this possibility, single neuron recordings in adult zebrafish and goldfish tectum have both described that many tectal neurons have compound visual RFs comprised of multiple non-overlapping regions of visual space (Schellart et al., 1979; Sajovic and Levinthal, 1982).

Convergence at the TL-PyrN circuit could additionally enhance PyrN visual sensitivity compared to SMTLs. If all SMTL inputs that converge on a PyrN convey similar information, for example OFF responses with highly overlapping RFs, the activity patterns in these PyrN TL inputs would be highly synchronized. If the noise in these input channels is independent and non-correlated, convergence of these inputs onto the same dendrite would increase the signal-to-noise ratio of the PyrN response. This is due to the fact that the signal would scale linearly in proportion to input number, whereas the noise would scale in proportion to the square root of input number (Faisal et al., 2008). In addition, highly synchronous inputs onto a dendrite can result in supralinear synaptic integration. In other systems, synchronous inputs to distal dendrites of pyramidal neurons can sum to generate dendritic spikes that propagate to the cell body to drive spiking (Gasparini et al., 2004; Losonczy and Magee, 2006). Due to the integrative properties of dendrites, a stimulus evoking a weak, but highly synchronous response in TL neurons could lead to a stronger response in the post-synaptic PyrN through both passive (linear summation) and active (dendritic spiking) forms of dendritic integration. However, the degree of synchrony between neurons in TL has not been examined experimentally and it is unknown whether synchronous inputs to SM dendrites can initiate regenerative spikes in tectal PyrN dendrites. Furthermore, it is unknown whether PyrN spiking requires synchronous input from both RGCs on their SFGS dendrite and SMTLs on their SM dendrite.

A variation on this model is one in which RGC input increases spike frequency in PyrNs, but coincident activation of RGC and TL inputs generates spike bursts. Tonic and burst firing modes have been demonstrated in thalamic relay cells (Sherman, 2001), hippocampal pyramidal neurons (Grienberger et al., 2014) and pyramidal cells in the hindbrain of weakly electric fish (Metzen et al., 2016). TL input may sensitize PyrN responsiveness to RGC input following detection of a change in global luminance. Such a “wake up call” mechanism has been proposed in visual thalamic circuits, where neuromodulatory inputs depolarize geniculate cells to facilitate burst firing, thereby maximizing the effect of retinal input activation (Sherman, 2001). Burst firing of ensembles of thalamocortical relay cells is thought to serve as a priming signal for downstream cortical columns. If PyrNs serve an equivalent role in the fish tectum, a luminance change in the visual field could serve as a “wake up call” for a large population of PyrNs. However, this assumes that visually responsive PyrNs receive input from visually responsive TL neurons. An alternative scenario is one in which visually responsive PyrNs receive input from TL neurons encoding saccadic eye movements. Within this model, saccade-related signals converging onto SM PyrN dendrites could serve as the “wake up call,” activating PyrNs in tectum in response to spontaneous eye movements as has previously been proposed (Northmore, 2017). In both of these models a dynamic visual environment, generated by either saccadic scene shifts or changes in scene luminance, could lead to increased PyrN activity in tectum. Acetylcholine release by PyrNs could function to convert changes in the visual scene into a priming signal for downstream tectal circuits processing location-specific information, such as prey item tracking (Semmelhack et al., 2014; Bianco and Engert, 2015) or predator detection (Temizer et al., 2015; Dunn et al., 2016). Consistent with this model, strong modulation of retinotectal neurotransmission by acetylcholine has been previously demonstrated in goldfish tectum (Schmidt and Freeman, 1980; Langdon and Freeman, 1987; King and Schmidt, 1991).

In this study we identified the *hspGGFF23C* transgenic as a tool to specifically label neurons in the larval zebrafish TL that project to the SM layer of tectal neuropil: SMTLs. Genetic access will enable future studies employing cell type-specific *in vivo* functional imaging of SMTLs labeled with genetically encoded calcium or voltage sensors. These experiments will be able to directly examine if TL inputs to PyrNs convey visual or saccade-related information. Genetic targeting of SMTLs will also facilitate loss-of-function experiments using either focal laser ablation or chemogenetic methods (Curado et al., 2007) to reduce SMTL input to PyrNs. Together these studies will shed light on how TL input shapes visual processing in tectum to influence ecologically relevant visual behaviors.

## Materials and Methods

### Transgenic Fish

Zebrafish adults and larvae were maintained at 28°C on a 14/10 h light/dark cycle.

*Tg(id2b:Gal4-VP16)mpn215, Tg(hspGGFF23C), Tg(HuC:lynTagRFP-t)mpn404, Tg(UAS-E1B:NTR-mCherry), TgBAC(slc17a6b:LOXP-DsRed-LOXP-GFP)nns14Tg*, and *Tg(-17.6isl2b:TagRFP)zc80* transgenic lines have been previously described (Davison et al., 2007; Asakawa et al., 2008; Poulain and Chien, 2013; Satou et al., 2013; Förster et al., 2017). All larvae used were double mutants for *mitfa*^−/−^ *(nacre)* and *roy*^−/−^. All animal procedures conformed to the institutional guidelines of the M the Purdue University Institutional Animal Care and Use Committee (IACUC).

### Immunohistochemistry

VGluT1 antibody staining was performed as previously described (DeMarco et al., 2019). Briefly, anesthetized larvae were fixed overnight in a solution of 4% paraformaldehyde/4% sucrose in PBS. For antigen retrieval, fish were heated to 63°C for 15 min in 150 mM Tris-HCl. Following antigen retrieval larvae were incubated in blocking solution (5% donkey serum in PBS with 0.1% Triton-X and 1% DMSO) for 1–2 h prior to primary antibody incubation for 1–2 days in blocking solution. Primary antibody used: rabbit anti-VGluT1 (Abcam Cat #ab77822, RRID:AB_2187677) and chicken anti-GFP (GeneTex Cat# 13970, RRID:AB_371416). Secondary antibody incubation was overnight in blocking solution. Secondary antibodies used: Goat anti-chicken AlexaFluor 488 (Invitrogen Cat#A11039, RRID:AB_2534096) and Goat anti-rabbit AlexaFluor 555 (Invitrogen Cat# A21430, RRID:AB_2535849).

### Statistical Analysis

Data sets were analyzed using GraphPad Prism software version 801 (GraphPad Software, Inc., La Jolla, CA). All data displayed a normal distribution (*p* ≥ 0.05). One-way ANOVA was used to identify differences among means for data sets with three or more groups and Tukey's *post-hoc* test was used for comparisons. *P* < 0.05 were considered significant. Dunnett's adjusted *p*-values are displayed in the figures and the Results section. Graphs and table show mean ± SEM for each group.

### Embryo Injections

Genetic mosaic labeling of single PyrNs was performed by expression of the membrane targeted EGFP plasmid, 4xnrUAS:EGFP-caax (a gift from B. Appel and J. Hines, University of Colorado, Denver, CO), along with RNA encoding Tol2 transposase into *Tg(id2b:Gal4VP16,UAS-E1B:NTR-mCherry)* double transgenic embryos. DNA construct was pressure-injected at a concentration of 25–50 ng/μl into one- to eight-cell-stage embryos. For *PSD95-EGFP* labeling, a 50 ng/μl solution of 14UAS PSD95:GFP 5UAS DSRedExpress (Addgene Plasmid #74315; RRID:Addgene_74315) was pressure injected into *Tg(id2b:Gal4VP16)* transgenic embryos.

### Image Acquisition

For live confocal imaging between 6 and 8 dpf larvae were anesthetized in 0.016% tricaine and embedded in 2% low-melting-point agarose. Imaging was performed on a Nikon C2 confocal microscope equipped with solid state lasers for excitation of EGFP (488 nm) and mCherry/TagRFP (555 nm). Whole-larvae imaging was performed using a Nikon Plan Fluor 4 × 0.1 NA air objective using 5 μm z-steps. Whole-brain imaging of live larvae and immunofluorescently stained larvae was performed using a Nikon LWD 16 × 0.8 NA water immersion objective using 1–1.5 μm z-steps. For *PSD95-EGFP* imaging, single neurons were imaged using a Nikon 60 × 1.0 NA water immersion objective. Optical sections were acquired using 0.6–1 μm z-steps. For VGluT1 immunofluorescence imaging, single neurons were imaged using a Nikon 40 × 1.15 NA water immersion objective. Optical sections were acquired using 0.3 μm z-steps.

### Image Processing

Image stacks were visualized and analyzed using ImageJ FIJI software (http://fiji.sc/Fiji). 3D rendering was performed using the 3D Viewer FIJI plugin (Schmid et al., 2010). To generate subvolumes containing single PyrN arbors (SM, SFGS, or SGC) the 3D clipping tool in the 3D Viewer FIJI plugin was used to remove all other cell regions. Skeletonized tracings were generated using the semi-automated neurite segmentation plugin Simple Neurite Tracer in FIJI (Longair et al., 2011). For measurements of retinotopic area, image volumes were rotated −40° about the Y-axis using the ImageJ “3D Project” plugin, subsequently the areas (of PyrN arbors and neuropil labeling in different transgenic lines) were measured by manually drawing a convex polygon around the manually thresholded label image.

### Colocalization Analysis and 3D Cell Counting

To analyze colocalization between two image channels, stacks were analyzed using JACoP, a co-localization analysis plugin for ImageJ FIJI software. As many colocalization metrics can be sensitive to background noise, all image stacks were first background subtracted using a rolling ball of a 50-pixel radius. Fluorescently labeled regions were thresholded semi-autonomously using the 3D Iterative Threshold from the 3D ImageJ Suite with a constant minimum-threshold and volume range. Cell overlap was calculated through JACoP's object-based methods using a 3D center-of-mass based approach with a constant minimum and maximum object size. Data is reported as a number of colocalizing-couples paired with total number of identifiable cells.

Colocalization was further assessed using JACoP via Pearson's correlation coefficient (PCC), a metric which calculates the linear correlation between pixel intensities contained in both channels. Further analysis was performed using Van Steensel's cross-correlation coefficient (CCF). This metric is generated by translating one image along the x-axis while recalculating the PCC at each new location. In the event of positive correlation, a relative maximum should be observed at ΔX = 0 with values decreasing toward a PCC of 0 in either lateral direction.

## Data Availability Statement

The raw data supporting the conclusions of this article will be made available by the authors, without undue reservation.

## Ethics Statement

This animal study was reviewed and approved by Purdue University Institutional Animal Care and Use Committee.

## Author Contributions

ER, ED, AT, and BH performed the experiments. ER and AT analyzed the data. KK shared unpublished reagents. ER wrote the manuscript. All authors contributed to the manuscript and approved the submitted version.

## Conflict of Interest

The authors declare that the research was conducted in the absence of any commercial or financial relationships that could be construed as a potential conflict of interest.

## Abbreviations

CB: cerebellum
HB: hindbrain
MB: midbrain
OT: optic tectum
PyrN: pyramidal neuron
RF: receptive field
RGC: retinal ganglion cell
SAC: stratum album centrale
SFGS: stratum fibrosum et griseum superficiale
SGC: stratum griseum centrale
SM: stratum marginale
SMTL: stratum marginale-projecting TL neuron
SO: stratum opticum
SPV: stratum periventriculare
TL: torus longitudinalis
VGluT1: vesicular glutamate transporter 1
VGluT2b: vesicular glutamate transporter 2b.
